# Dispensability of Tubulin Acetylation for 15-protofilament Microtubule Formation in the Mammalian Cochlea

**DOI:** 10.1247/csf.20057

**Published:** 2021-01-20

**Authors:** Justine Renauld, Nicolas Thelen, Odile Bartholomé, Brigitte Malgrange, Marc Thiry

**Affiliations:** 1 GIGA-Neurosciences, Cell Biology Unit, University of Liège, Liège, Belgium; 2 Department of Otolaryngology, Case Western Reserve University, Cleveland, OH, United States of America; 3 GIGA-Neurosciences, Developmental Neurobiology Unit, University of Liège, Liège, Belgium

**Keywords:** Acetylation, cytoskeleton, microtubule, inner ear, supporting cells

## Abstract

The development of hearing in mammals requires the formation and maturation of a highly organized and specialized epithelium known as the organ of Corti. This epithelium contains two types of cells, the sensory cells, which are the true receptors of auditory information, and the surrounding supporting cells, which are composed of a highly developed cytoskeleton essential to the architecture of the mature organ of Corti. The supporting cells are the only mammalian cells reported to contain the unusual 15-protofilament microtubules. In this paper, we show that 15-protofilament microtubules appear between the second and fourth day after birth in the pillar cells of the organ of Corti in mice. We also show that contrary to what has been described in the nematode worm *Caenorhabiditis. elegans*, microtubule acetylation is not essential for the formation of 15-protofilament microtubules in mice but is required for fine-tuning of their diameter.

## Introduction

The development of hearing requires the formation and maturation of a highly organized and specialized epithelium known as the organ of Corti (Roth and Bruns, 1992). Sound vibrations cause the displacement of fluid within the inner ear, which results in the movement of mechanosensory hair cells in the organ of Corti. The deflection of hair cell stereocilia causes depolarization and triggers the release of the excitatory neurotransmitter glutamate, resulting in the transduction of auditory vibration signals into an electrical signal that is sent through the auditory nerve to the brain (Pickles, 2015).

The cytoskeletal elements of the organ of Corti, which include an abundant amount of actin filaments within the stereocilia of the sensory cells as well as microtubules within the surrounding supporting cells, have been studied for decades for their importance in developing and maintaining the precise morphology of the organ required for hearing (Andrade, 2015; Henderson *et al.*, 1994; Zetes *et al.*, 2012). The mature pillar cells are characterized by an abundant and highly organized cytoskeleton framework that confers stiffness that protects the cells from mechanical deformation, which occurs each time a sound wave is transmitted to the inner ear (Henderson *et al.*, 1995; Tolomeo and Holley, 1997). The opening of the tunnel of Corti is an essential step in the maturation of the hearing organ, in which the supporting cells increase their length and detach from each other. This allows the intercellular space to be filled with the vibration-conducting cochlear fluid (Ito *et al.*, 1995). Furthermore, an absence of this triangular open space between the pillar cells has been shown to be a potential reason for hearing loss. Indeed, mutation of the gap junction connexin 26, the most common cause of congenital sensorineural hearing loss, has been linked with the maturation of pillar cells. The downregulation of connexin 26 before the opening of the tunnel of Corti decreases the number of microtubules within the pillar cells. This has been associated with the failure of the tunnel of Corti to open and resultant deafness (Chen *et al.*, 2018).

The unique characteristic of pillar microtubules is that they are composed of 15 protofilaments (pf) instead of the common 13 pf encountered in mammalian cells (Kikuchi *et al.*, 1991; Tucker *et al.*, 1992). Several papers already show the relationship between 15-pf microtubules and large bundle stability (Chalfie and Thomson, 1982; Topalidou *et al.*, 2012). Furthermore, other researchers have already established the correlation between tubulin isotype, posttranslational modification such as acetylation and pf number (Cueva *et al.*, 2012; Savage *et al.*, 1989; Ti *et al.*, 2018). The presence of unusual pf numbers per microtubule has been previously reported in invertebrates. For example, it has been shown that in the moth *Heliothis viriscens*, the expression of a specific β-tubulin isotype (β2) is responsible for the formation of 16-pf microtubules contained within spermatid axonemes (Raff, 1997). Interestingly, this same study reported that the expression of this particular β-tubulin isotype in *Drosophila* leads to the formation of 16-pf microtubules instead of the 13-pf microtubules usually present in the same subset of microtubules of the axoneme. Additionally, *C. elegans* were used to show the essential role of certain isoforms of alpha or beta tubulin in the formation of unusual microtubules and their functional role in touch sensitivity (Bounoutas *et al.*, 2009; Fukushige *et al.*, 1999; Savage *et al.*, 1989; Wade, 2009). In subsequent invertebrate studies, acetylation of microtubules was implicated in the formation of microtubules with 15 pf (Cueva *et al.*, 2012; Topalidou *et al.*, 2012). However, the occurrence of distinct morphological changes and the importance of various tubulin isotypes or their posttranslational modifications is less well reported in vertebrates.

In this work, we studied the fine structure of microtubules in pillar cells of the organ of Corti in mice. We were interested in the early stages after birth, between P0 and P25, a critical period in the development of hearing. We also examined the number of pf in microtubules in pillar cells in a mutant mouse depleted for acetyltransferase Atat1, an enzyme known to acetylate alpha tubulins. Our results reveal that the microtubules of the pillar cells consist of 13 pf in the early postnatal stage and rapidly acquire 15 pf from P4 onwards. We also show that the formation of microtubules with 15 pf is not affected by tubulin acetylation, contrary to what has been described in invertebrates.

## Materials and Methods

### Mice

Animal handling was carried out in compliance with the University of Liege Animal Care and Use Committee guidelines. The NMRI mice were bred in our animal facility.

Acetyl transferase *Atat1* mutant mice come from ‘The Knockout Mouse Project’ referenced as *Atat1*^tm1(KOMP)Vlcg^. Heterozygous mice are used for reproduction and offspring are genotyped by PCR as previously reported (Kim *et al.*, 2013). The day of birth was recorded as post-natal day 0 (P0). NMRI mice were sacrificed at different time points from P0 to P30. Forceps were used to dissect the cochleae under a stereomicroscope. The cochlear apex was carefully pierced to allow rapid penetration of the fixative.

### Immunohistochemistry

The cochleae were fixed in a solution composed of 2% formaldehyde in PBS (140 mM NaCl, 2.7 mM KCl, 1.5 mM KH_2_PO_4_, 16 mM Na_2_HPO_4_, pH 7.4) for 1 h at 4°C. After 3 washes at 4°C in PBS, the cochleae were decalcified at 4°C in 4% (w/v) EDTA in PBS as long as necessary. The samples were then washed several times in PBS and incubated at 4°C in 30% (w/v) sucrose in PBS on a gently rotating platform until the cochleae sank in the tube. The cochleae were embedded in 7.5% (w/v) gelatin and 15% (w/v) sucrose in PBS for 15 min at 37°C. The preparation was finally plunged into an isopentane bath on dry ice for solidification. The cryosections (12 μm thick) were obtained by a cryostat (Microm HM 560, Prosan).

The cryosections were rinsed in PBS. The sections were permeabilized and blocked in PGT (PBS – 0.25% gelatin- 0.3% triton X100 ) for 30 min. Then, the sections were incubated with the primary antibody solution diluted in PGT overnight at 4°C (1/10 000 - mouse anti-acetylated alpha-tubulin antibody - T6793, Sigma-Aldrich; 1/150 - rabbit anti-myosin VI antibody - M5187, Sigma-Aldrich). The sections were washed in PBS and incubated for 30 min at 37°C with the secondary antibody diluted in PBS (1/250 - goat anti-rabbit Alexa 594 antibody - R37117, Thermo Fisher Scientific; 1/250 - goat anti-mouse Alexa 488 antibody - A-11001, Thermo Fisher Scientific). After being washed in PBS, the nuclei were stained by incubating the sections with DAPI (1: 50,000, 4',6-diamidino-2-phenylindole dihydrochloride, Sigma, St Louis, USA) at 37°C for 15 min. Finally, the cryosections were rinsed in PBS and mounted with Citifluor AF1 (Laborimpex, Brussels, Belgium).

The mid- and basal turns of the cochlea were analyzed. The slides were examined under an Olympus FV1000 confocal microscope using a 60x objective. The optical sections were analyzed with the FV10-ASW 1.7 viewer software.

### Electron microscopy

The cochleae were fixed under vacuum for 1 h at room temperature in 2.5% glutaraldehyde in 0.1 M Sörensen’s buffer pH 7.4. After 3 washes in Sörensen’s buffer, the samples were permeabilized under vacuum with 2% digitonin during 1 to 2 hours at room temperature. The cochleae were washed in Sörensen’s buffer before being put in contact with 4% tannic acid in Sorensen pH 6.8 for an hour at room temperature under vacuum. This additional step allows the visualization of the number of pf (adaptation of the tannic acid protocol developed by Tilney in 1973 (Tilney *et al.*, 1973)). After 3 washes, the adult cochleae were decalcified in 4% EDTA. The samples were then post-fixed for 60 min with 2% osmium tetroxide in Sörensen’s buffer pH 7.4, washed in deionized water, dehydrated at room temperature through a graded ethanol series (70, 96 and 100%), and embedded in Epon for 48 h at 60°C. Semithin (1 μm thick) and ultrathin sections (50 nm thick) were obtained by means of an ultramicrotome (Reichert Ultracut E) equipped with a diamond knife (Diatome). Ultrathin sections were mounted on copper grids and were contrasted with uranyl acetate and lead citrate for 15 min each (Reynolds, 1963).

Semithin sections were put on a glass slide and stained with 1% toluidine blue in sodium tetraborate (1% solution, Na_2_B_4_O_7_·H_2_O) and then covered with a coverslip sealed with Entellan® (Merck, Overijse, Belgium). The slides were examined under a Zeiss Axioplan microscope. Ultrathin sections were examined with a Jeol JEM- 1400 transmission electron microscope at 80 kV and photographed with a 11 MegaPixel bottom-mounted TEM camera system (Quemesa, Olympus). The images were analyzed via iTEM software (Olympus SIS).

### Quantifications and statistical analysis

The measurement of the diameter was taken from the center of a pf to the center of the pf located on the opposite side. If the microtubule was not perfectly circular, the smallest diameter was measured to avoid variability due to oblique sections through the microtubules. One-way analysis of variance statistical test followed by Tukey-Kramer test was performed using the software Past 4 software (Hammer *et al.*, 2001). In order to compare the microtubule diameter in WT and Atat1 KO mice, unpaired t-test and F-test have been used with the same software.

### Analyses of microtubule diameter

Each condition was analyzed as follows: 3 animals per condition for P2, P4 and P30 pillar cells, 6 pictures per animal, 3 random microtubules measured per pictures. 3 animals for the kinocilium measurement, 2 pictures per animal, 4 random microtubules measured per picture.

### Analyses between WT and *Atat1* KO mice

Only microtubules present in a bundle were analyzed. Each condition was analyzed as follows: 3 animals per condition,10 pictures per animal, 5 random microtubules measured per picture.

### Analyses of microtubule pf number and density (Fig. S1)

For the analysis of microtubule pf, 11 microtubules with clearly distinguishable pf were selected in inner pillar cell of each condition. The density was calculated on ultrathin section of pillar cell medial part by dividing the total number of microtubules by the area of the cell.

## Results

### The number of pf in microtubules of the pillar cells as well as their diameter changes during the development of the auditory organ

**Fig. 1:**
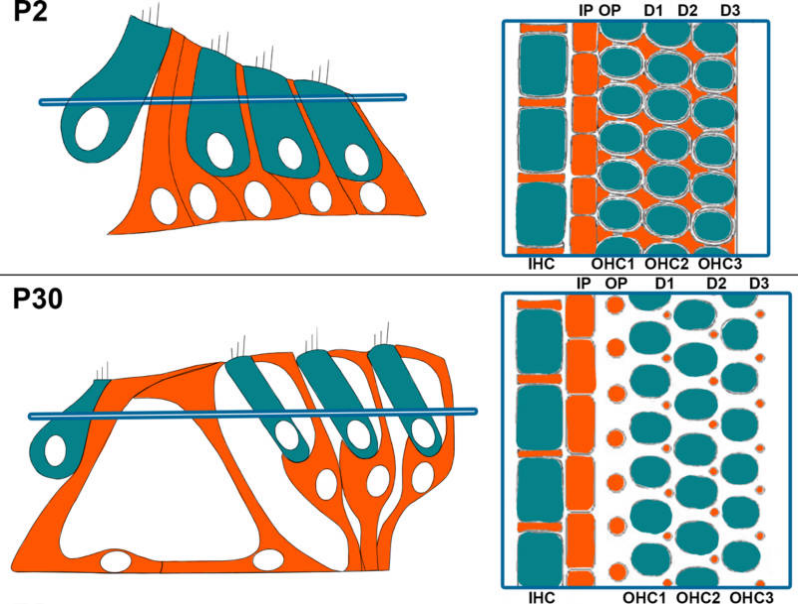
Schematic representation of the organization of the organ of Corti. Transverse (left) and orthogonal view (right) at P2 (top) or P30 (bottom). The section plan is indicated in blue. IHC: inner hair cell; Ip: inner pillar cell; OHC: outer hair cell; Op: outer pillar cell; D1 to D3: Deiters’ cell 1 to 3.

**Fig. 2:**
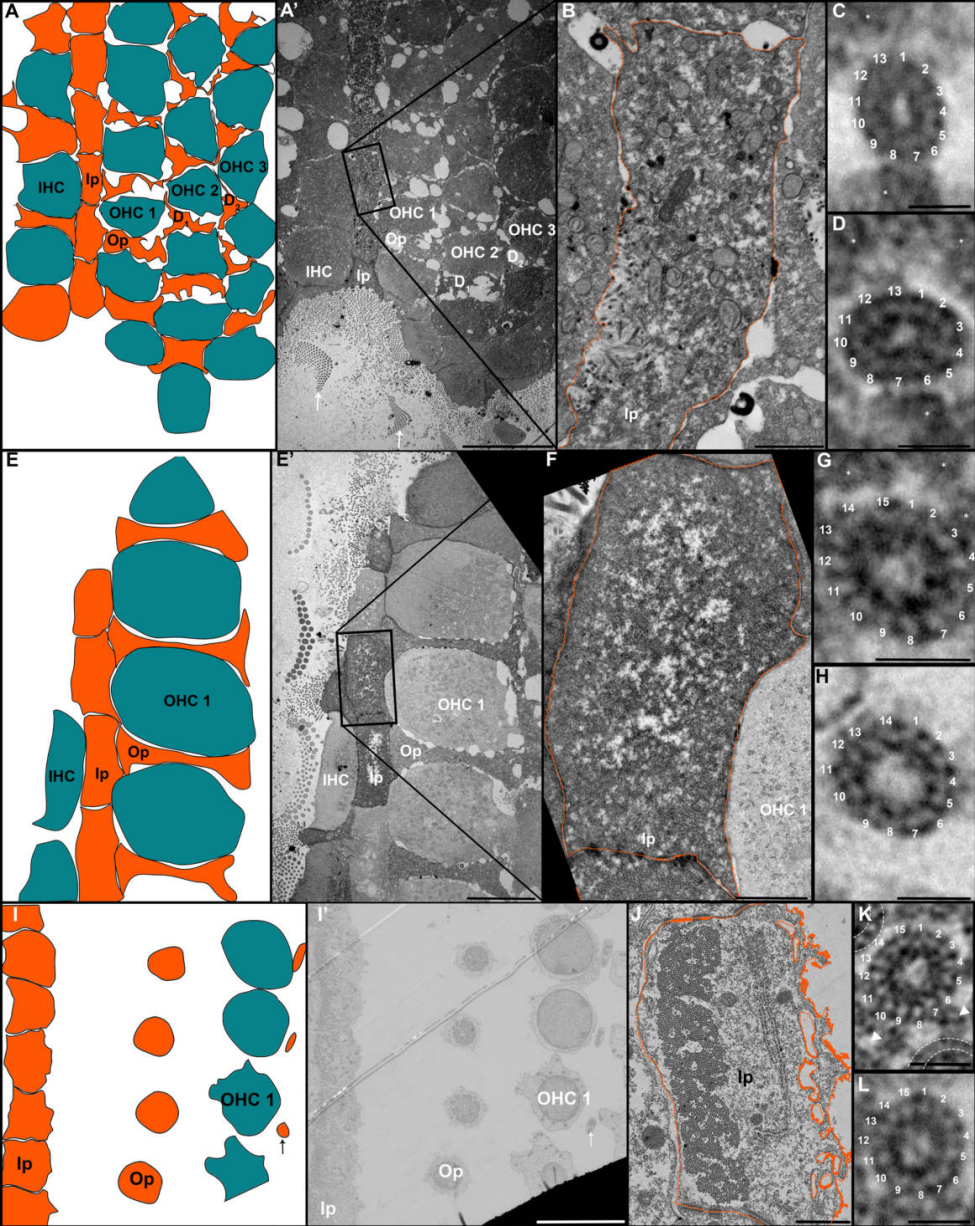
Increase in the number of microtubule protofilaments during the development of the inner pillar cells of the hearing organ. Schematic representation at P2 (A) , P4 (E) and P30 (I). General view at P2 (A’), P4 (E’) and P30 (I’). Detail of the cytoplasm of an inner pillar cell at P2 (B), P4 (F) and P30 (J). High magnification of individual microtubules at P2 (C and D), P4 (G and H) and at P30 (K and L). IHC: inner hair cell; Ip: inner pillar cell; OHC 1 to 3: outer hair cell 1 to 3; Op: outer pillar cell; D1 to 2: Deiters’ cell 1 to 2. The arrows in panel A’ indicate the stereocilia of sensory cells. The arrow in panel I’ indicates the phalangeal process of a Deiters’ cell. The arrowheads in panel K indicate dense material linking microtubule together. Asterisks indicate electron dense materials in panels C, D, G. Dashed lines indicated adjacent microtubules in panel K. Scale bars: 10 μm (A’, E’ and I’), 1 μm (B, F and J) and 20 nm (C, D, G , H , K and L).

We examined the morphology of microtubules present in pillar cells between P2 and P30 by transmission electron microscopy as seen in Fig. 1. Tannic acid was used in the sample preparation to better visualize the pf. Transverse sections of pillar cells at P2 (Fig. 2A-A’) show few microtubules, surrounded by electron dense material (Fig. 2B). A high magnification image of microtubules containing 13 pf is seen (Fig. 2C-D). At P4, the microtubules occupy a larger surface area of the cytoplasm of the pillar cells (Fig. S1). The electron-dense material observed at P2 is still present (Fig. 2G asterisks). At higher magnification, we identify microtubules at 14 and 15 pf (Fig. 2G and 2H). On cross sections of pillar cells at P25 (Fig. 2I and 2I’), the number of microtubules is higher compared to the previous stages (Fig. S1). They form a compact bundle within a thinned cytoplasm, across the entire width of the cells (Fig. 2J). The microtubule bundle is organized in a very regular geometric structure in which the microtubules are connected by dense material (Fig. 2K arrowheads). At this stage, the microtubules consist of 15 pf (Fig. 2K and 2L).

**Fig. 3:**
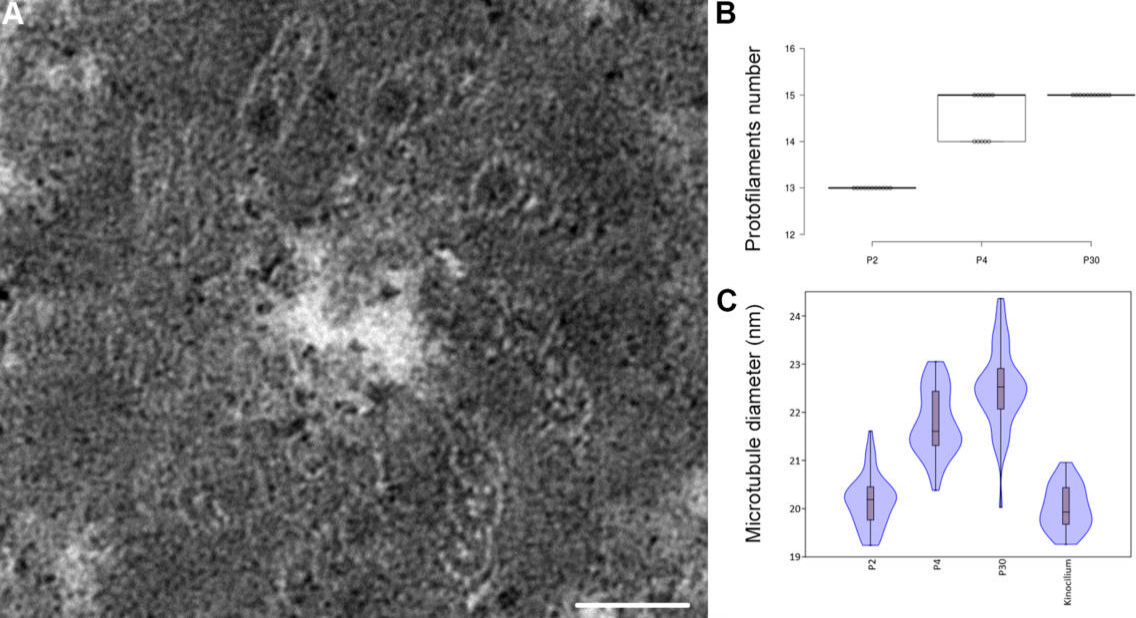
Increase in the diameter of the microtubules during the development of the inner pillar cells of the hearing organ. Ultrastructure of the basal body of a kinocilium in hair cell (A), and representation of the number of protofilament per microtubule of supporting cells at P2, P4 and P30 (B). Violin plot comparing the average diameter of microtubules present in supporting cells at P2, P4 and P30 and microtubules in the kinocilium (C). Scale bar: 50 nm.

The number of protofilaments per microtubule increase from 13 at P2 to 15 at P30, with an intermediate stage of 14 and 15pf at P4 (Fig. 3B). Due to the low number of microtubules at P2 and the difficulty of slicing exactly perpendicular sections in order to count the number of pf, we also measured the diameter of each microtubule in pillar cells to compare the microtubules between P2 and P30. The microtubules present in the kinocilium of sensory cells were used at P2 (Fig. 3A) to act as a 13 pf control. Statistical analysis indicated a significantly smaller diameter of the microtubules in kinocilium in comparison to those present in P4 and the mature pillar cells (Fig. 3C). We repeated this measurement between early pillar cells (P2 and P4) and mature pillar cells (P30), and found a smaller diameter in the microtubules present in pillar cells at P2 and P4 compared to those present at P30 (Fig. 3C and Table I). The average diameter of the microtubules present in early pillar cells is comparable to the diameters seen in kinocilium, confirming the presence of 13 pf at P2. To assess the increase of microtubule numbers during the development, their number and density per μm^2^ was analyzed in the medial part of the inner pillar cell from P2 to P30. Even if they are not evenly distributed in the cytoplasm, the density of microtubule increases from 8,88/μm^2^ at P2 to 173,56/μm^2^ at P30 (Fig. S1). Those data are in accordance with previously published data in which the number of microtubules and density increase during development of pillar cells (Szarama *et al.*, 2012; Tucker *et al.*, 1992).

### Acetylation of microtubules in pillar cells is not necessary for 15-pf microtubules

**Fig. 4:**
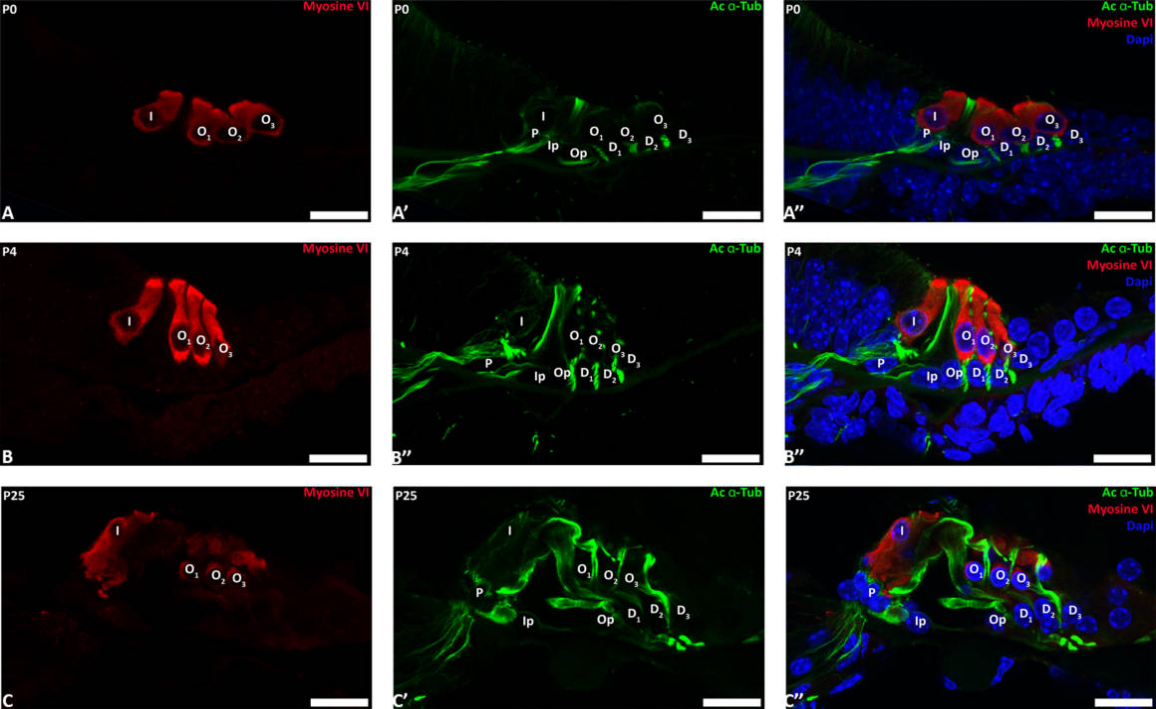
Acetylation of microtubule tubulins of pillar cells between P0 and P25. Confocal immunostaining of P0 (A), P4 (B) and P25 cochlear sections. Acetylated α-tubulin is labeled in green, myosin VI is labelled in red (sensory cells labeling). Nucleus are labelled by DAPI (Blue). I: inner hair cell; Ip: inner pillar cell; O 1 to 3: outer hair cell 1 to 3; Op: outer pillar cell; D1 to D3: Deiters’ cell 1 to 3; P: phalangeal cell. Scale bars: 20 μm.

**Fig. 5:**
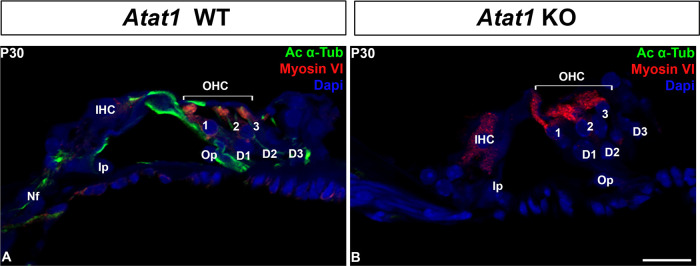
Absence of acetylated microtubules in the mutant *Atat1* mouse. Immunostaining of section of organ of Corti in WT (A) and *Atat1* KO at P30. Acetylated α-tubulin is labeled in green, myosin VI is labelled in red (sensory cells labeling). Nucleus are labelled by DAPI (Blue). I: inner hair cell; Ip: inner pillar cell; Nf: Nerve fibers; O 1 to 3: outer hair cell 1 to 3; Op: outer pillar cell; D1 to D3: Deiters’ cell 1 to 3; P: phalangeal cell. Scale bars: 20 μm.

**Fig. 6:**
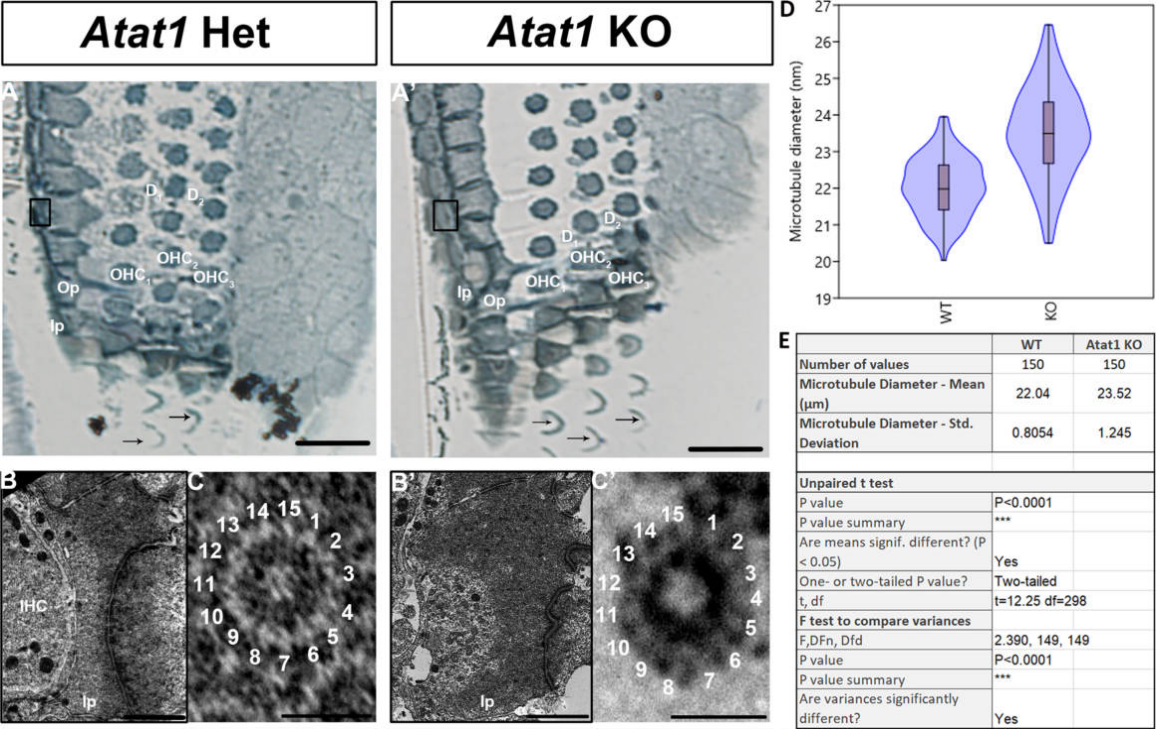
Tubulin acetylation is not required for 15-pf microtubule formation in mice. Semithin section of the organ of Corti (A-A’). Ultrastructure of pillar cells at P30 (B-B’). High magnification of individual microtubules (C-C’). Violin and box plot of the mean of the diameter of pillar cells’ microtubules (D) and associated statistical analysis (E). IHC: inner hair cell; Ip: inner pillar cell; OHC: outer hair cell; Op: outer pillar cell; D1 to D2: Deiters’ cell 1 to 2. The arrows indicate the stereocilia of sensory cells. Scale bars: 50 μm (A-A’), 2 μm (B-B’) and 20 nm (C-C’).

Acetylation of tubulins is implicated in the modification of the number of pf in invertebrates by a mechanism that is not yet fully understood (Cueva *et al.*, 2012; Topalidou *et al.*, 2012). Previous studies stipulated that acetylation is necessary but not sufficient for the formation of 15-pf microtubules, suggesting that this enzyme plays an indirect role in the formation of those unusual microtubules (Howes *et al.*, 2014; Li and Yang, 2015). To analyze the implication of this post-translational modification in the development of the organ of Corti, we first analyzed this process in the pillar cells of the organ of Corti at P0, P4 and P25 (Fig. 4A-B-C). The acetylation of microtubules in pillar cells is present at their apex at P0 (Fig. 4A) and extends in the longitudinal axis at P4 (Fig. 4B). The microtubule bundle is acetylated at P25 from the apex to the base of the cell (Fig. 4C). Previous work has shown that this acetylation is the result of the acetyltransferase ATAT1 enzyme (Kalebic *et al.*, 2013). In this context, we analyzed the acetylation of the microtubules in a mouse model deleted for this enzyme. First, in order to verify that the mutant mouse depleted for acetyltransferase ATAT1 no longer contains acetylated alpha tubulin we applied immunofluorescence labelling with an anti-acetylated alpha tubulin antibody. At P30, we observed no labelling in the organ of Corti of the mutant mouse unlike the wild-type mouse, attesting to the absence of acetylation of alpha tubulins in the mutant devoid of the ATAT1 enzyme (Fig. 5A-B). Next, we analyzed the morphology of the organ of Corti in WT or heterozygous and *Atat1* KO mice at P30 on semi-thin section (Fig. 6A-B). The general morphology of the organ of Corti, such as its cell organization, the size of the pillar cell or the tunnel of Corti appeared unaffected by the absence of tubulin acetylation. We then analyzed the morphology and the diameter of microtubules at higher magnification (Fig. 6C, D and E). The number of pf per microtubule is 15 for *Atat1* Heterozygous and KO mice (Fig. 6C and D). Interestingly, the average diameter of the microtubules of pillar cells is larger and more variable in *Atat1* KO compared to Atat1 Heterozygous, as seen in the Violin plot (Fig. 6D and E) (Mean (SD): Heterozygous 22,0376 nm (0.805) n=150; *Atat1* KO: 23,5211 nm (1,2451) n=150). Unpaired t test : P<0.0001 ; F test for variance P<0.0001.

## Discussion

In this study, we analyzed the morphology of microtubules in the organ of Corti during development in mice. In the adult organ of Corti, the number of pf is 15 in pillar cells. In vertebrates, those supporting cells are the only ones that do not have the usual 13-pf configuration (Kikuchi *et al.*, 1991; Tucker *et al.*, 1992). The function of those unusual 15-pf microtubules in the hearing process is still unknown, though it has been proposed that the 15-pf structure provides rigidity (Chaaban and Brouhard, 2017). In the worm *C. elegans*, several studies have shown that the loss of 15-pf structure can lead to loss of function of the mechanoreceptor (Bounoutas *et al.*, 2009). This suggests that mutations or loss of this pf arrangement may play a role in hearing loss or other pathologies.

One known difference between the 13- and 15-pf configuration is the disappearance of the seam running along the length of the microtubule (Li *et al.*, 2002; Sui and Downing, 2010). In this situation, the microtubules adopt a conformation in which each α-tubulin is located next to another α-tubulin of the adjacent pf. It has been hypothesized that this seam gives a line of weakness and could act as a trigger point for disassembly (Ayoub *et al.*, 2015; Katsuki *et al.*, 2014; Wade, 2009). Therefore, in the organ of Corti, this may suggest that the existence of those 15-pf microtubules may fulfill the functional needs of those supporting cells and give stability to the cell. In this study, we showed that the number of pf increase from the 13 to 15 pf early in development, around P4. This early modification is made before the hearing onset and could be a prerequisite to the correct function of the organ of Corti. This is further supported by the fact that these cells are situated between the sensory cells and the basilar membrane and play a key role in the propagation of sound waves. At P25, we also observed well-organized bundles of microtubules in which the microtubules are linked together by electron-dense material that could correspond to cross-linking proteins. This organization also provides resistance to bending and stretching (Liew *et al.*, 2015; Tolomeo and Holley, 1997). Until now, no study has shown the impact of the absence of those unusual 15-pf microtubules on auditory function. This is because it is unknown how to specifically alter the 15-pf microtubules in mammals.

Given the dispensability of tubulin acetylation in the formation of 15-pf microtubules in pillar cells, the tubulin isotypes could be another means of regulating the number of pf in microtubules in vertebrates. In fact, it was previously shown that β-tubulin isotypes play an important role in the architecture of microtubules in invertebrates (Meurer-Grob *et al.*, 2001). The presence of an unusual pf number per microtubule were extensively studied in invertebrates such as Drosophila, in which the presence of a specific moth beta-tubulin isotype can induce the formation of these unusual 15-pf microtubules (Raff, 1997). This result showed that this β-tubulin isotype is sufficient to induce the formation of 16-pf microtubules. In this study, we showed that 15-pf microtubules appear between P2 and P4, which correspond to the appearance of the expression of βV-tubulin isotype. In this regard, it would be interesting to analyze the role of the βV-tubulin isotype in the formation of 15-pf microtubules, as our previous research showed that this isotype is only expressed in the pillar and Deiters’ cells of the organ of Corti (Renauld *et al.*, 2015). Our previous publication on the spatiotemporal dynamic of β-tubulin isotypes during the development of the organ of Corti highlighted the specific pattern of the β-V-tubulin that appears at a key stage of the supporting cells’ development. The correlation between the timing and localization of the 15-pf microtubules and the expression of this isotype make it an interesting candidate for the formation of 15-pf microtubules.

One mechanism expected to control microtubule function is the post-translational modification of their tubulin subunits (Janke, 2014; Janke and Montagnac, 2017). The acetylation of the α-tubulin constrains the 15-pf number in the worm, *C. elegans* (Cueva *et al.*, 2012). Here, we demonstrated that the acetylation was dispensable to create or maintain 15-pf microtubules inside the organ of Corti, as we did not find any difference in the number of pf between the WT and the *Atat1* KO mice. In *C. elegans*, the acetylation of the α-tubulin was a determinant for the formation of those unusual microtubules and also for their number and organization (Topalidou *et al.*, 2012). Interestingly, despite the constant 15-pf microtubules, we also observed that microtubules varied in diameter, as observed in *C. elegans* (Topalidou *et al.*, 2012). This greater variation in diameter in *Atat1* KO mice could be explain by the model proposed by Cueva in which the acetylation of α-tubulin stabilizes interprotofilament salt bridges between adjacent α-tubulins (Cueva *et al.*, 2012). Cueva stipulated that the acetylation of the α-tubulin disrupt an intramonomere salt bridge to create an intermonomere salt bridge instead. This new intermonomere salt bridge would change the lateral interactions between α-tubulins leading to an interprotofilament angle in favor of 15 pf. In this study of the pillar cells, the absence of acetylation would increase the range of value of the interprotofilament angle which would increase the diameter variability, but other parameter, such as the nature of the tubulin isoforms, the microtubule associated proteins or other posttranslational modifications would restrain this angle enough to maintain the number of pf constant even if the diameter changes. It should therefore be interesting to study all these parameters in order to understand which ones impose the formation of microtubules at 15 pf.

Furthermore, *in vitro* research demonstrated that the acetylation itself of the lysine in position 40 of the α-tubulin had no significant effect on microtubule structure (Howes *et al.*, 2014). For this reason, in 2015, Li and his coworker hypothesized the acetylation of the α-tubulin may form a specific docking site for an unidentified protein. This unidentified protein may then promote the formation of 15-pf microtubules (Li and Yang, 2015).

In addition to the isotype of tubulin, acetylation is probably not the only factor influencing the number of pf in vertebrates. Several studies have shown that some microtubules associated proteins (MAPs) like doublecortin or kinesin promote the formation of 13-pf microtubules (Akhmanova and Severin, 2004; Howes *et al.*, 2014; Moores *et al.*, 2004). It would not be surprising to discover some MAPs that promote 15-pf microtubules in pillar cells.

The study showing the absence of physiological difference in the *Atat1* KO hearing published in 2013 was surprising for us as we were expecting a more important role of this enzyme in hearing based on previous data in zebrafish (Kim *et al.*, 2013). In the zebrafish model, the inhibition of this enzyme leads to developmental defects such as shortening of the body, neuromuscular defects, or reduction of eye and head size (Akella *et al.*, 2010). Furthermore, abundant numbers of acetylated microtubules were reported in the cochlea in pillar and Deiter’s cells (Li and Yang, 2015; Szarama *et al.*, 2012; Tannenbaum and Slepecky, 1997) as observed in our immunofluorescence labeling for acetylated tubulin, and ATAT1 as the enzyme responsible for the acetylation of the tubulin in mice (Kalebic *et al.*, 2013; Liu *et al.*, 2018; Shida *et al.*, 2010). We observed the disappearance of the acetylated microtubules in the pillar cells of the *Atat1* KO mice, confirming the hypothesis that this enzyme is responsible of the acetylation of the microtubules present in pillar cells. This result showed that the inhibition of tubulin acetylation doesn’t lead to any developmental defects in mammalian cells which contain a large amount of acetylated microtubules in contrast to the zebrafish study (Akella *et al.*, 2010). Other studies performed on *Chlamydomonas reinhardtii* and *Tetrahymenas thermophile* showed that the modification of α-tubulin to prevent its acetylation didn’t create any phenotype in those models (Gaertig *et al.*, 1995; Kozminski *et al.*, 1993). It’s important to note that when the enzyme responsible for the deacetylation of microtubules is absent, resulting in hyper-acetylation of microtubules, there is no major alteration in the phenotype of mice either. This data seems to show a high tolerance in the level of acetylation of microtubules in mice (Zhang *et al.*, 2008). This tubulin acetylation may not be essential to the development of organisms but could play a role in their adaptation to their environment (Janke and Montagnac, 2017).

In conclusion, we show for the first time that 15-pf microtubules appear between the second and fourth day after birth in the mouse pillar cells. We also show that contrary to what has been observed in the nematode worm *C. elegans*, the acetylation of those microtubules is not essential for the formation of the 15-pf microtubule structure, even if we see an increase in diameter variability. Further mutational research would be necessary to confirm if one of the tubulin isotypes expressed in those cells leads to this 15-pf microtubule production and the role of this specific microtubule structure in hearing development.

## Contribution

J.R., N.T and M.T designed the study. J.R. performed many of the experiments. N.T. and O.B. measured the microtubule diameter. B.M. shared Atat1 animal colonies. J.R and N.T. contributed to data interpretation and J.R wrote the manuscript with input from all coauthors.

## Figures and Tables

**Table I:** Statistical analysis of microtubules diameter within the inner pillar cell and the kinocilium of the hearing organ in mice: Tukey’s multiple comparison test performed between each sample.

	P2	P4	P30	Kinocilium
Number of values	54	54	54	24
Microtubule Diameter - Mean (μm)	20.16	21.78	22.5	20.02
Microtubule Diameter - Std. Deviation	0.5286	0.6749	0.8185	0.4597
Tukey’s Multiple Comparison Test	Mean Diff.	q	Significant? P<0.05?	Summary
P2 vs P4	–1.629	18.14	Yes	***
P2 vs P30	–2.347	26.13	Yes	***
P2 vs Kinocilium	0.1351	1.18	No	ns
P4 vs P30	–0.7181	7.994	Yes	***
P4 vs Kinocilium	1.764	15.41	Yes	***
P30 vs Kinocilium	2.483	21.68	Yes	***

## Abbreviations

MAPs: microtubules associated proteins
Pf: protofilament
P: postnatal day

## References

[B1] Akella, J.S., Wloga, D., Kim, J., Starostina, N.G., Lyons-Abbott, S., Morrissette, N.S., Dougan, S.T., Kipreos, E.T., and Gaertig, J. 2010. MEC-17 is an α-tubulin acetyltransferase. Nature, 467: 218–222. 20829795 10.1038/nature09324 PMC2938957

[B2] Akhmanova, A. and Severin, F. 2004. Thirteen is the lucky number for doublecortin. Dev. Cell, 7: 5–6. 15239948 10.1016/j.devcel.2004.06.011

[B3] Andrade, L.R. 2015. Evidence for changes in beta- and gamma-actin proportions during inner ear hair cell life. Cytoskeleton, 72: 282–291. 26033950 10.1002/cm.21227 PMC4529820

[B4] Ayoub, A.T., Klobukowski, M., and Tuszynski, J.A. 2015. Detailed Per-residue Energetic Analysis Explains the Driving Force for Microtubule Disassembly. PLoS Comput. Biol., 11: 1–21. 10.1371/journal.pcbi.1004313 PMC4452272 26030285

[B5] Bounoutas, A., O’Hagan, R., and Chalfie, M. 2009. The Multipurpose 15-Protofilament Microtubules in C. elegans Have Specific Roles in Mechanosensation. Curr. Biol., 19: 1362–1367. 19615905 10.1016/j.cub.2009.06.036 PMC2757273

[B6] Chaaban, S. and Brouhard, G.J. 2017. A microtubule bestiary: Structural diversity in tubulin polymers. Mol. Biol. Cell, 28: 2924–2931. 29084910 10.1091/mbc.E16-05-0271 PMC5662251

[B7] Chalfie, M. and Thomson, J.N. 1982. Structural and functional diversity in the neuronal microtubules of Caenorhabditis elegans. J. Cell Biol., 93: 15–23. 7068753 10.1083/jcb.93.1.15 PMC2112106

[B8] Chen, S., Xie, L., Xu, K., Cao, H.-Y., Wu, X., Xu, X.-X., Sun, Y., and Kong, W.-J. 2018. Developmental abnormalities in supporting cell phalangeal processes and cytoskeleton in the Gjb2 knockdown mouse model. Dis. Model. Mech., 11: dmm033019. 29361521 10.1242/dmm.033019 PMC5894950

[B9] Cueva, J.G., Hsin, J., Huang, K.C., Goodman, M.B., and Cueva et al. 2012. Posttranslational Acetylation of α-Tubulin Constrains Protofilament Number in Native Microtubules. Curr. Biol., 22: 1066–1074. 22658592 10.1016/j.cub.2012.05.012 PMC3670109

[B10] Fukushige, T., Siddiqui, Z.K., Chou, M., Culotti, J.G., Gogonea, C.B., Siddiqui, S.S., and Hamelin, M. 1999. MEC-12, an alpha-tubulin required for touch sensitivity in C. elegans. J. Cell Sci., 112 (Pt 3): 395–403. 9885292 10.1242/jcs.112.3.395

[B11] Gaertig, J., Cruz, M.A., Bowen, J., Gu, L., Pennock, D.G., and Gorovsky, M.A. 1995. Acetylation of lysine 40 in a-tubulin is not essential in Tetrahymena thermophila. J. Cell Biol., 129: 1301–1310. 7775576 10.1083/jcb.129.5.1301 PMC2120470

[B12] Hammer, Ø., Harper, D.A.T., and Paul D. Ryan. 2001. Past: Paleontological statistics software package for education and data analysis. Palaeontol. Electron., 4: 1–9.

[B13] Henderson, C.G., Tucker, J.B., Chaplin, M. a, Mackie, J.B., Maidment, S.N., Mogensen, M.M., and Paton, C.C. 1994. Reorganization of the centrosome and associated microtubules during the morphogenesis of a mouse cochlear epithelial cell. J. Cell Sci., 107 (Pt 2): 589–600. 8207081

[B14] Henderson, C.G., Tucker, J.B., Mogensen, M.M., Mackie, J.B., Chaplin, M.a, Slepecky, N.B., and Leckie, L.M. 1995. Three microtubule-organizing centres collaborate in a mouse cochlear epithelial cell during supracellularly coordinated control of microtubule positioning. J. Cell Sci., 108 (Pt 1): 37–50. 7738112 10.1242/jcs.108.1.37

[B15] Howes, S.C., Alushin, G.M., Shida, T., Nachury, M.V., and Nogales, E. 2014. Effects of tubulin acetylation and tubulin acetyltransferase binding on microtubule structure. Mol. Biol. Cell, 25: 257–266. 24227885 10.1091/mbc.E13-07-0387 PMC3890346

[B16] Ito, M., Spicer, S.S., and Schulte, B.a. 1995. Cytological changes related to maturation of the organ of Corti and opening of Corti’s tunnel. Hear. Res., 88: 107–123. 8575987 10.1016/0378-5955(95)00106-e

[B17] Janke, C. 2014. The tubulin code: Molecular components, readout mechanisms, functions. J. Cell Biol., 206: 461–472. 25135932 10.1083/jcb.201406055 PMC4137062

[B18] Janke, C. and Montagnac, G. 2017. Causes and Consequences of Microtubule Acetylation. Curr. Biol., 27: R1287–R1292. 29207274 10.1016/j.cub.2017.10.044

[B19] Kalebic, N., Sorrentino, S., Perlas, E., Bolasco, G., Martinez, C., and Heppenstall, P.A. 2013. αTAT1 is the major α-tubulin acetyltransferase in mice. Nat. Commun., 4: 1962. 23748901 10.1038/ncomms2962

[B20] Katsuki, M., Drummond, D.R., and Cross, R.A. 2014. Ectopic A-lattice seams destabilize microtubules. Nat. Commun., 5: 3094. 24463734 10.1038/ncomms4094 PMC3921467

[B21] Kikuchi, T., Takasaka, T., Tonosaki, A., Katori, Y., and Shinkawa, H. 1991. Microtubules of guinea pig cochlear epithelial cells. Acta Otolaryngol., 111: 286–290. 2068915 10.3109/00016489109137389

[B22] Kim, G.-W., Li, L., Gorbani, M., You, L., and Yang, X.-J. 2013. Mice Lacking α-Tubulin Acetyltransferase 1 Are Viable but Display α-Tubulin Acetylation Deficiency and Dentate Gyrus Distortion. J. Biol. Chem., 288: 20334–20350. 23720746 10.1074/jbc.M113.464792 PMC3711300

[B23] Kozminski, K.G., Diener, D.R., and Rosenbaum, J.L. 1993. High level expression of nonacetylatable alpha-tubulin in Chlamydomonas reinhardtii. Cell Motil. Cytoskeleton, 25: 158–170. 7686822 10.1002/cm.970250205

[B24] Li, H., DeRosier, D.J., Nicholson, W.V., Nogales, E., and Downing, K.H. 2002. Microtubule structure at 8 A resolution. Structure, 10: 1317–1328. 12377118 10.1016/s0969-2126(02)00827-4

[B25] Li, L. and Yang, X.-J. 2015. Tubulin acetylation: responsible enzymes, biological functions and human diseases. Cell. Mol. Life Sci., 72: 4237–4255. 26227334 10.1007/s00018-015-2000-5 PMC11113413

[B26] Liew, K.M., Xiang, P., and Zhang, L.W. 2015. Mechanical properties and characteristics of microtubules: A review. Compos. Struct., 123: 98–108.

[B27] Liu, W., Wang, C., Yu, H., Liu, S., and Yang, J. 2018. Expression of acetylated tubulin in the postnatal developing mouse cochlea. Eur. J. Histochem., 62: 2942. 30088716 10.4081/ejh.2018.2942 PMC6119817

[B28] Meurer-Grob, P., Kasparian, J., and Wade, R.H. 2001. Microtubule structure at improved resolution. Biochemistry, 40: 8000–8008. 11434769 10.1021/bi010343p

[B29] Moores, C. a, Perderiset, M., Francis, F., Chelly, J., Houdusse, A., and Milligan, R.a. 2004. Mechanism of microtubule stabilization by doublecortin. Mol. Cell, 14: 833–839. 15200960 10.1016/j.molcel.2004.06.009

[B30] Pickles, J.O. 2015. *Auditory pathways: Anatomy and physiology*, 1st ed. 10.1016/B978-0-444-62630-1.00001-9 25726260

[B31] Raff, E.C. 1997. Microtubule Architecture Specified by a beta -Tubulin Isoform. Science (80-.)., 275: 70–73. 10.1126/science.275.5296.70 8974394

[B32] Renauld, J., Johnen, N., Thelen, N., Cloes, M., and Thiry, M. 2015. Spatio-temporal dynamics of β-tubulin isotypes during the development of the sensory auditory organ in rat. Histochem. Cell Biol., 144: 403–416. 26210854 10.1007/s00418-015-1350-2

[B33] Reynolds, E.S. 1963. The use of lead citrate at high pH as an electron-opaque stain in electron microscopy. J. Cell Biol., 17: 208–212. 13986422 10.1083/jcb.17.1.208 PMC2106263

[B34] Roth, B. and Bruns, V. 1992. Postnatal development of the rat organ of Corti II. Hair cell receptors and their supporting elements. Anat. Embryol. (Berl)., 185: 571–581. 1605368 10.1007/BF00185616

[B35] Savage, C., Hamelin, M., Culotti, J.G., Coulson, a, Albertson, D.G., and Chalfie, M. 1989. mec-7 is a beta-tubulin gene required for the production of 15-protofilament microtubules in Caenorhabditis elegans. Genes Dev., 3: 870–881. 2744465 10.1101/gad.3.6.870

[B36] Shida, T., Cueva, J.G., Xu, Z., Goodman, M.B., and Nachury, M.V. 2010. The major α-tubulin K40 acetyltransferase αTAT1 promotes rapid ciliogenesis and efficient mechanosensation. Proc. Natl. Acad. Sci. USA, 107: 21517–21522. 21068373 10.1073/pnas.1013728107 PMC3003046

[B37] Sui, H. and Downing, K.H. 2010. Structural basis of interprotofilament interaction and lateral deformation of microtubules. Structure, 18: 1022–1031. 20696402 10.1016/j.str.2010.05.010 PMC2976607

[B38] Szarama, K.B., Gavara, N., Petralia, R.S., Kelley, M.W., and Chadwick, R.S. 2012. Cytoskeletal changes in actin and microtubules underlie the developing surface mechanical properties of sensory and supporting cells in the mouse cochlea. Development, 139: 2187–2197. 22573615 10.1242/dev.073734 PMC3357912

[B39] Tannenbaum, J. and Slepecky, N.B. 1997. Localization of microtubules containing posttranslationally modified tubulin in cochlear epithelial cells during development. Cell Motil. Cytoskeleton, 38: 146–162. 9331219 10.1002/(SICI)1097-0169(1997)38:2<146::AID-CM4>3.0.CO;2-5

[B40] Ti, S.C., Alushin, G.M., and Kapoor, T.M. 2018. Human β-Tubulin Isotypes Can Regulate Microtubule Protofilament Number and Stability. Dev. Cell, 47: 175-190.e5. 30245156 10.1016/j.devcel.2018.08.014 PMC6362463

[B41] Tilney, L.G., Bryan, J., Bush, D.J., Fujiwara, K., Mooseker, M.S., Murphy, D.B., and Snyder, D.H. 1973. Microtubules: evidence for 13 protofilaments. J. Cell Biol., 59: 267–275. 4805001 10.1083/jcb.59.2.267 PMC2109099

[B42] Tolomeo, J.A. and Holley, M.C. 1997. Mechanics of microtubule bundles in pillar cells from the inner ear. Biophys. J., 73: 2241–2247. 9336220 10.1016/S0006-3495(97)78255-9 PMC1181125

[B43] Topalidou, I., Keller, C., Kalebic, N., Nguyen, K.C.Q., Somhegyi, H., Politi, K.A., Heppenstall, P., Hall, D.H., and Chalfie, M. 2012. Genetically separable functions of the MEC-17 tubulin acetyltransferase affect microtubule organization. Curr. Biol., 22: 1057–1065. 22658602 10.1016/j.cub.2012.03.066 PMC3382010

[B45] Tucker, J.B., Paton, C.C., Richardson, G.P., Mogensen, M.M., and Russell, I.J. 1992. A cell surface-associated centrosomal layer of microtubule-organizing material in the inner pillar cell of the mouse cochlea. J. Cell Sci., 102 (Pt 2): 215–226. 1400629 10.1242/jcs.102.2.215

[B47] Wade, R.H. 2009. On and around microtubules: an overview. Mol. Biotechnol., 43: 177–191. 19565362 10.1007/s12033-009-9193-5

[B48] Zetes, D.E., Tolomeo, J.a., and Holley, M.C. 2012. Structure and Mechanics of Supporting Cells in the Guinea Pig Organ of Corti. PLoS One, 7: e49338. 23145154 10.1371/journal.pone.0049338 PMC3492263

[B49] Zhang, Y., Kwon, S., Yamaguchi, T., Cubizolles, F., Rousseaux, S., Kneissel, M., Cao, C., Li, N., Cheng, H.-L., Chua, K., Lombard, D., Mizeracki, A., Matthias, G., Alt, F.W., Khochbin, S., and Matthias, P. 2008. Mice Lacking Histone Deacetylase 6 Have Hyperacetylated Tubulin but Are Viable and Develop Normally. Mol. Cell. Biol., 28: 1688–1701. 18180281 10.1128/MCB.01154-06 PMC2258784

